# Supplementary material for: The E3 ubiquitin ligase HectD3 attenuates cardiac hypertrophy and inflammation in mice
Source: Commun Biol. 2020 Oct 9;3:562. doi: 10.1038/s42003-020-01289-2 (PMC7547098; doi:10.1038/s42003-020-01289-2)
Supplement: Supplementary file 1 — Supplementary Information [file 42003_2020_1289_MOESM1_ESM.docx]

**Supplementary Figures with legends**

**
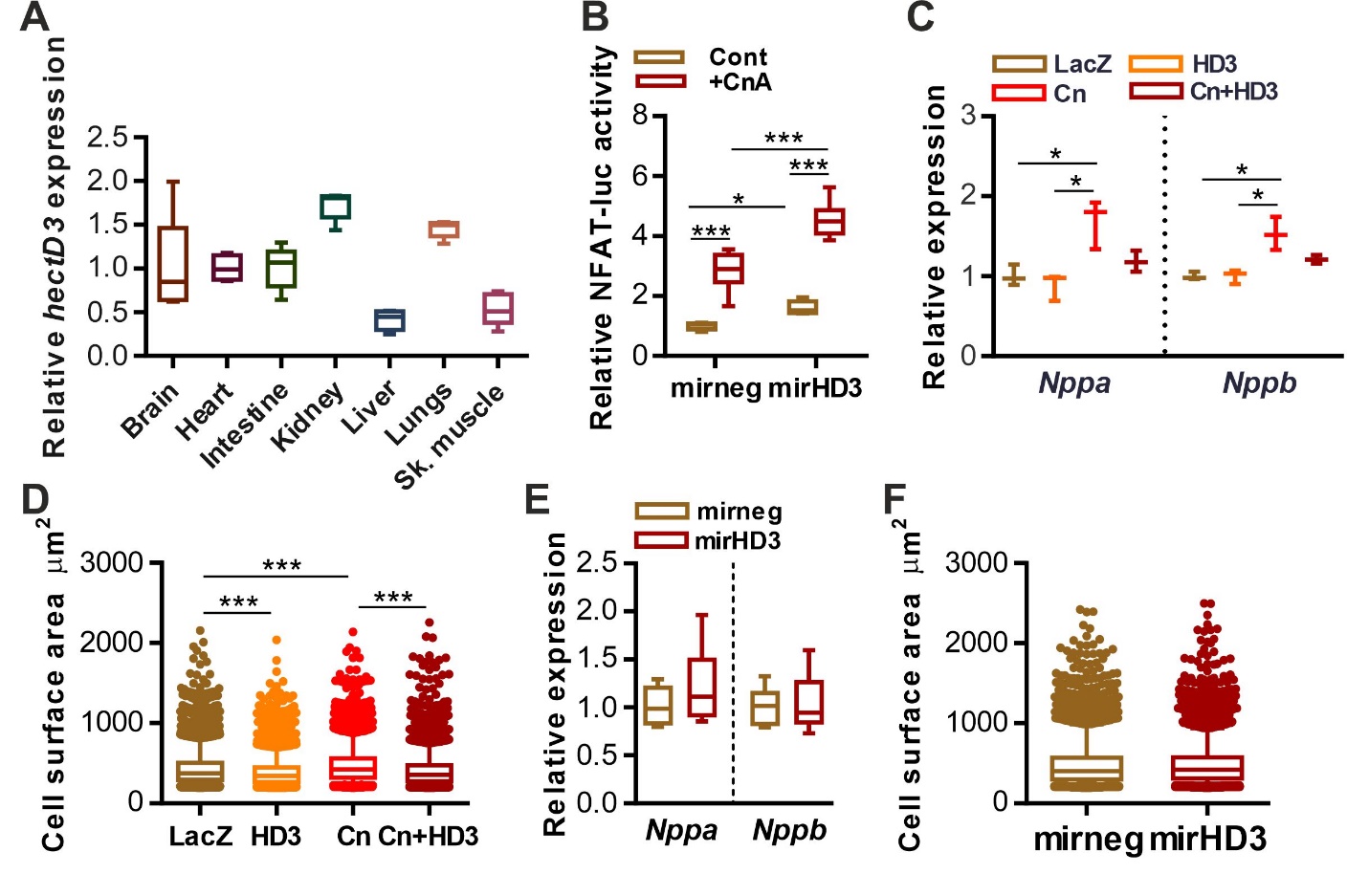
**

**Supplementary Figure 1: HectD3 interacts with and suppresses SUMO2 dependent activation of calcineurin-signaling and cardiomyocyte hypertrophy. A.** HectD3 expression levels in various tissues determined by quantitative real-time PCR (n=5 each). **B.** NFAT response element driven Firefly luciferase reporter assay was performed in NRVCMs to study the effect of knockdown of HectD3 (mirHD3), with or without constitutively active calcineurin A (CnA). Box plot indicates that HectD3 knockdown significantly induced luciferase activity, both, at baseline or in the presence of CnA. (n=12 each). **C.** Expression of fetal genes *nppa* and *nppb* determined by quantitative real-time PCR indicates downregulation of both the genes when HectD3 is overexpressed. (n=3 each). **D.** Cell surface area measurement of NRVCMs overexpressing CnA, HectD3, or both, indicates CnA increases cellular hypertrophy, whereas, the presence of HectD3 attenuated this activation. (n>1500 each). **E.** Expression of fetal genes *nppa* and *nppb* determined by quantitative real-time PCR indicates no effect of HectD3 knockdown on the both the genes. (n=6 each). **F.** Cell surface area measurement from NRVCMs where HectD3 was knocked-down indicated no significant differences compared to the control cells. (n>1500 each). mirneg (unrelated synthetic microRNA) was used as a control in all knockdown experiments. Statistical calculations were carried out two-tailed Students t-test or by one-way ANOVA. *: p<0.05, ***: p<0.001.

**
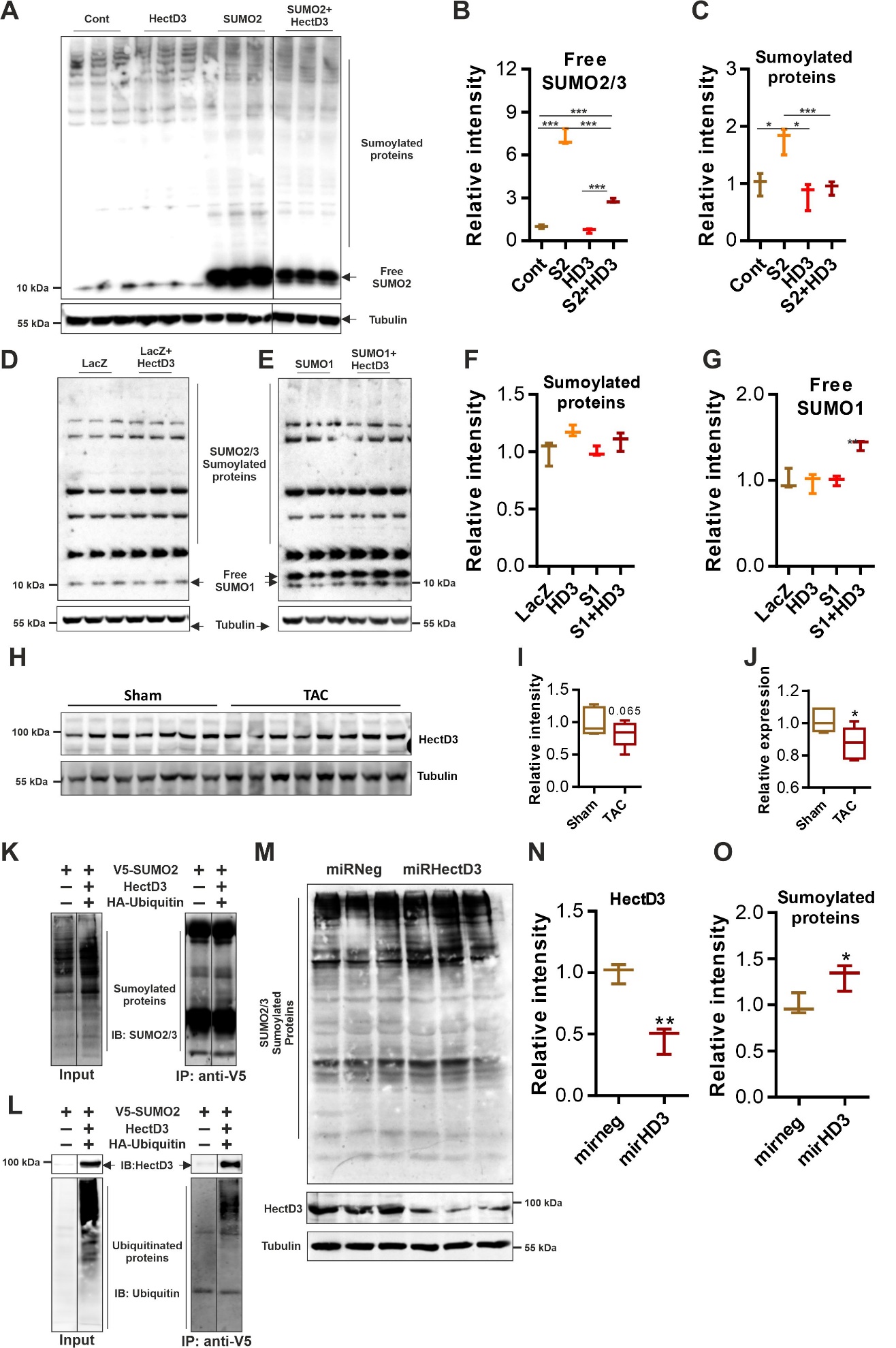
**

**Supplementary Figure 2: HectD3 regulates SUMO2-mediated sumoylation. A.** Immunoblot indicating the expression of SUMO2/3 and sumoylated proteins in HEK293A cells overexpressing SUMO2, HectD3, or both. **B** & **C** represents densitometry analysis of free SUMO2/3 or sumoylated proteins, respectively, shown in image **A**. (n=3 each). **D.** Immunoblot indicating the expression of SUMO1 and sumoylated proteins in NRVCMs overexpressing HectD3 or LacZ. **E.** Immunoblot indicating the expression of SUMO1 and sumoylated proteins in NRVCMs overexpressing SUMO1 alone or with HectD3. **F** & **G** represents the densitometry analysis of sumoylated proteins or free SUMO1 shown in image D and E, respectively. (n=4 each). Neither endogenous nor overexpressed SUMO1 was affected by HectD3 expression. NRVCMs expressing LacZ were used as a control. **H.** Immunoblots indicating HectD3 protein levels in mouse heart after pressure overload due to transverse aortic constriction (TAC). Its densitometry data is presented in (**I**) as a box plot**.** (n= 7 sham and 8 TAC). **J.** Quantitative real-time PCR data depicting significant downregulation of HectD3 transcript levels in mouse heart after TAC. (n= 7 sham and 8 TAC). **K.** Immunoblots indicating the input and IP blots where V5-tagged SUMO2 was either overexpressed alone or in the presence of both HectD3 (untagged) and ubiquitin (HA-tagged). IP was performed by anti-V5 magnetic beads and immunoblotting was carried out by anti-V5 antibody. **L.** Immunoblots indicating the input and IP samples showing HectD3 and ubiquitin detected by anti-HectD3 and anti-HA antibodies, respectively. Images show that anti-V5 beads could successfully pull down HectD3 as well as polyubiquitinated SUMO2 only in the condition where HectD3 and ubiquitin were expressed with SUMO2. **M.** Immunoblots indicating the expression of HectD3 and SUMO2/3-sumoylated proteins in NRVCMs where HectD3 was knocked-down using adenovirus encoding synthetic microRNA. **N.** Box plot with the densitometry analysis confirming ~50% downregulation of HectD3 after its knockdown. (n=3 each). **O.** Box plot representing densitometry analysis of SUMO2/3 sumoylated proteins shown in image **M** indicates that HectD3 knockdown led to moderate accumulation of SUMO2/3 sumoylated proteins. (n=3 each). Statistical calculations were carried out by two-tailed Student’s t-test. *: p<0.05, **: p<0.01, ***: p<0.001.

**
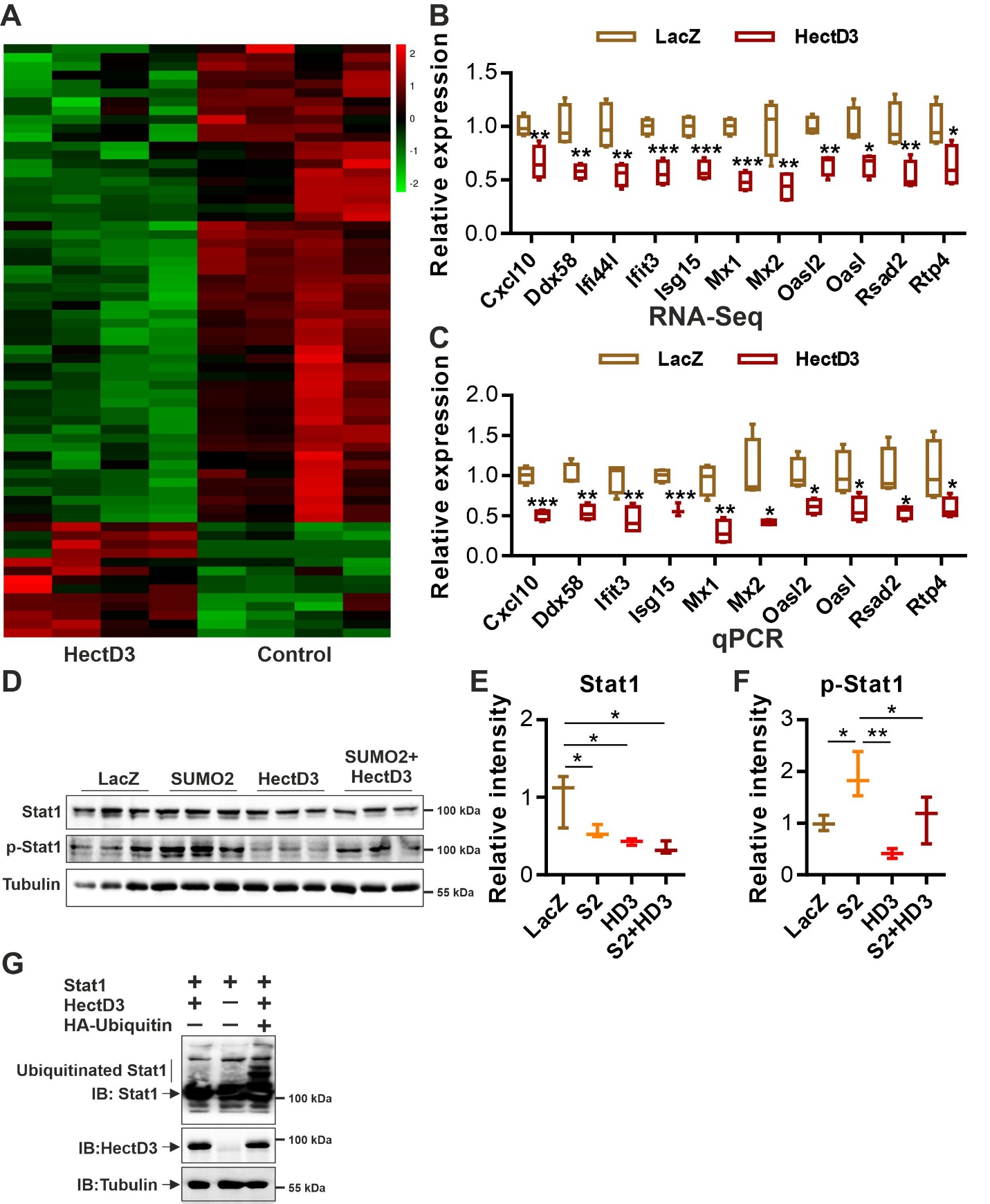
**

**Supplementary Figure 3: HectD3 targets interferon response proteins and Stat1 signaling in NRVCMs. A.** Heat map showing the differentially abundant genes in NRVCMs after HectD3 overexpression. **B.** Extracted FPKM values show the inhibitory effect of HectD3 overexpression on Stat1 transcription target genes (**B**), which could be further validated by quantitative real-time PCR (**C**). (n=4 each). **D.** Immunoblots displaying the expression of Stat1/p-Stat1 in LacZ, SUMO2, HectD3 and HectD3+SUMO2 overexpressing NRVCMs. Its densitometry data presented in **E**/**F** shows that SUMO2 overexpression significantly increased activation i.e. phosphorylation of Stat1. The presence of HectD3 however attenuated this activation. (n=3 each). **G.** *In vitro* ubiquitination assay exhibits poly-ubiquitination of Stat1 when HectD3 was overexpressed in tandem. Statistical calculations were carried out by two-tailed Student’s t-test or one-way ANOVA. *: p<0.05, **: p<0.01, ***: p<0.001.

**
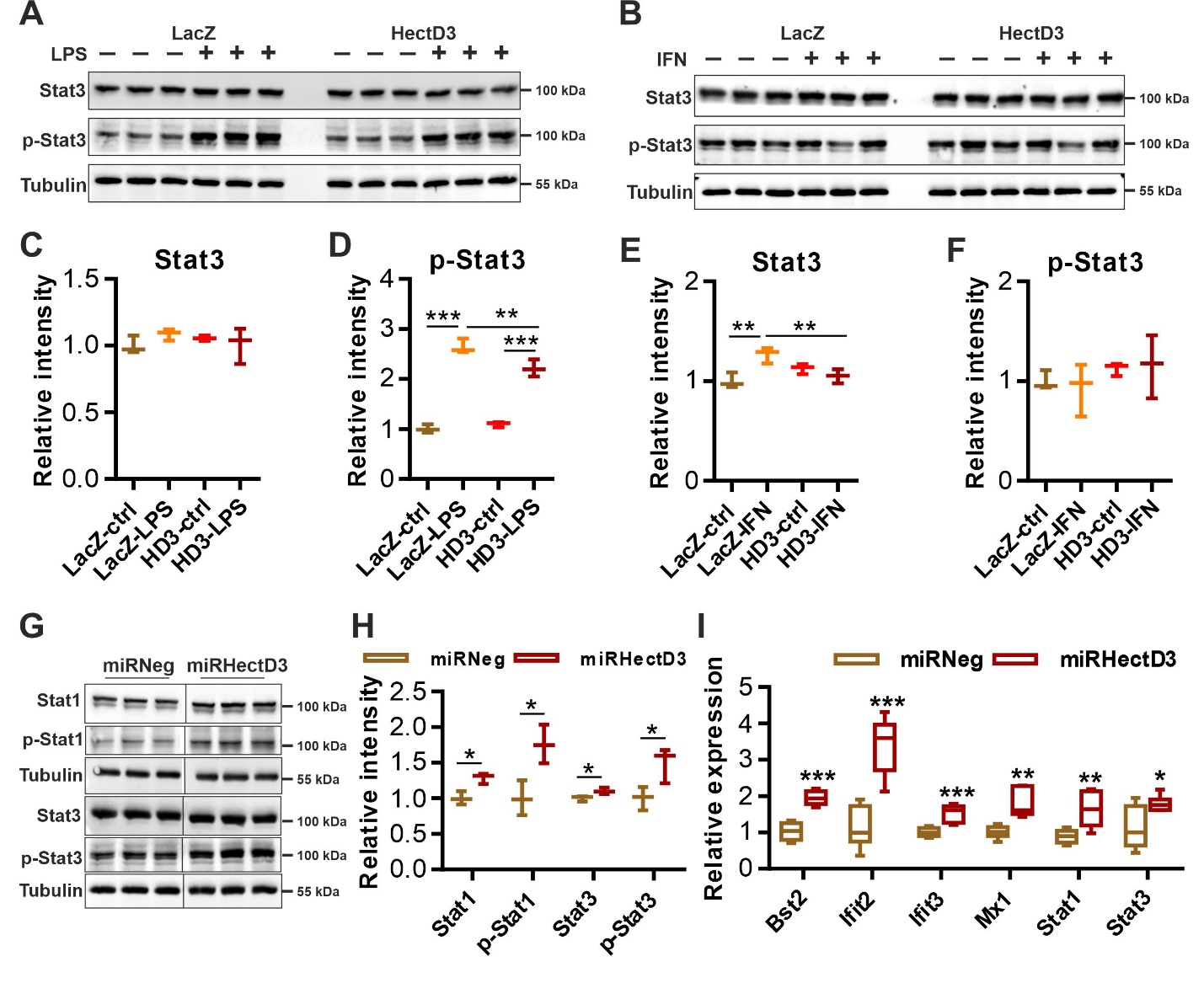
**

**Supplementary Figure 4: HectD3 inhibits LPS-/IFNγ-mediated activation of the inflammatory response in cardiomyocytes. A.** Immunoblots displaying the expression of Stat3/p-Stat3 in control and HectD3 overexpressing NRVCMs in the absence or the presence of LPS treatment. **B.** Immunoblots displaying the expression of Stat3/p-Stat3 in control and HectD3 overexpressing NRVCMs in the absence or the presence of IFNγ treatment. Densitometry analysis of **A** shown in **C**/**D** indicates that LPS significantly activated Stat3 by its phosphorylation, HectD3 overexpression however blunted this activation. Densitometry analysis of **B** shown in **E**/**F** indicates that IFNγ did not affect phosphorylation but only increased native Stat3 levels which were also again repressed by HectD3. (n=3 each). **G.** Immunoblots showing the expression of Stat1/p-Stat1 and Stat3/p-Stat3 under HectD3 knockdown conditions. **H.** Densitometry data from **G** presented as a box plot shows significant upregulation of Stat1/p-Stat1 and Stat3/p-Stat3 in NRVCMs when HectD3 was knocked-down. (n=3 each). **I.** Transcript levels of several interferon responsive Stat1-signaling genes determined by quantitative real-time PCR revealing the significant downregulation upon HectD3 knockdown. (n=6 each). Statistical calculations were carried out by two-tailed Student’s t-test or two-way ANOVA. *: p<0.05, **: p<0.01, ***: p<0.001.

**
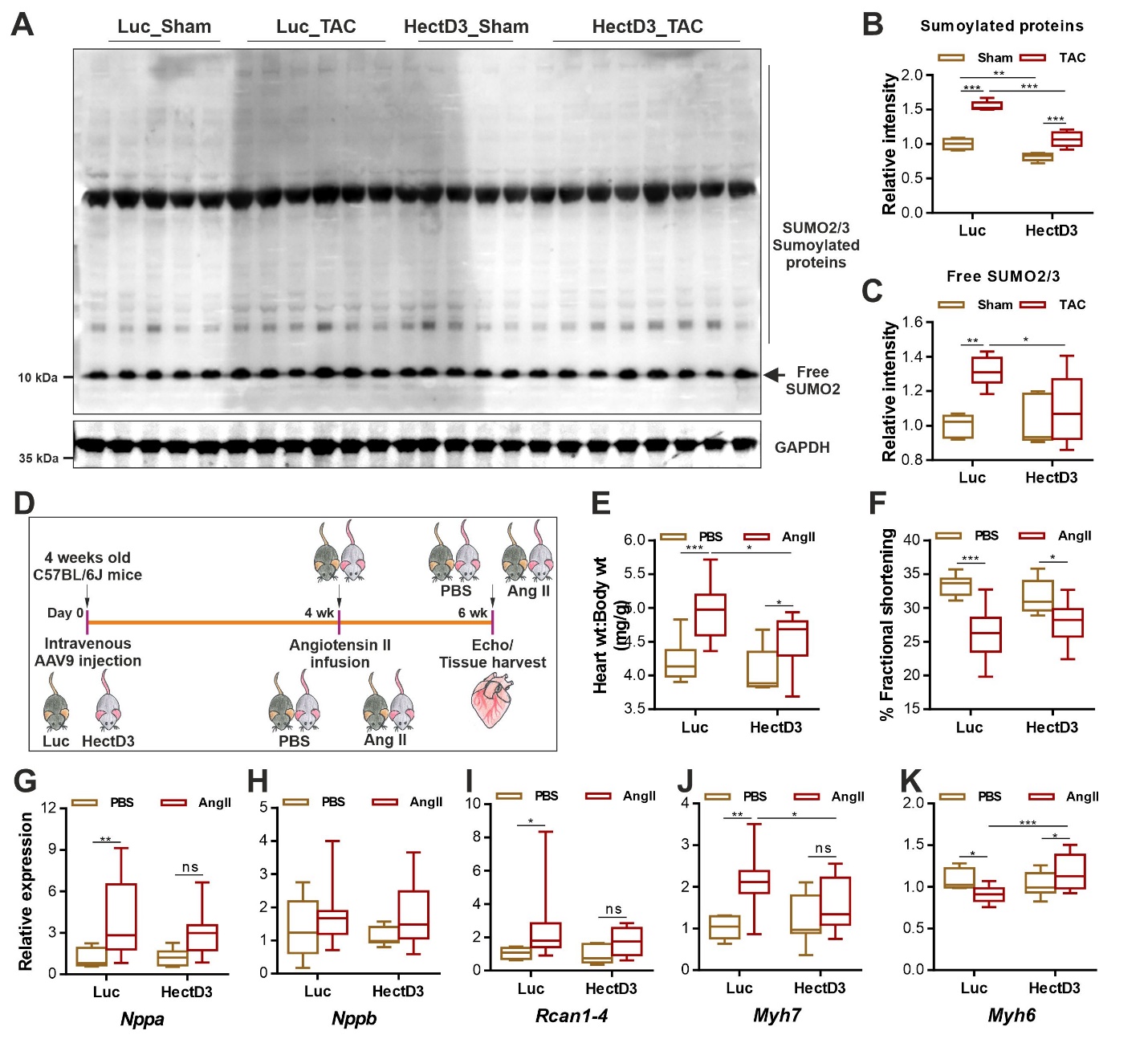
**

**Supplementary Figure 5: HectD3 suppresses SUMO2 dependent activation of calcineurin-signaling and cardiac hypertrophy. A.** Immunoblots indicating the proteins levels of SUMO2/3 and sumoylated proteins after transverse aortic constriction (TAC) or sham operations in mouse heart where HectD3 was overexpressed via AAV-mediated gene transfer. AAV-luciferase injected mice were used as control group. Its densitometry analysis indicates upregulation of sumoylated proteins (**B**) and free SUMO2/3 (**C**) after TAC. Increased expression of HectD3, however, significantly attenuated the upregulation of SUMO2/3 as well as sumoylated proteins. **D.** A self-explanatory outline of the experimental work-plan for AAV-mediated overexpression of HectD3 in mice, Angiotensin II (AngII) infusion and downstream experiments. **E.** Box plot indicating the heart weight (wt):body wt ratio in mice that were infused with AngII or PBS (control). Data indicates that AngII significantly increased this ratio in control mice, whereas, AAV9-mediated overexpression of HectD3 reduced the ratio. (n=6 (AAV-Luc PBS), 7 (AAV-HectD3 PBS), 11 (AAV-Luc AngII), 11 (AAV-HectD3 AngII)). **F.** Fractional shortening (%) was reduced after AngII treatment in both groups and HectD3 displayed no significant effect. (n=6 (AAV-Luc PBS), 7 (AAV-HectD3 PBS), 11 (AAV-Luc AngII), 11 (AAV-HectD3 AngII)). Expression of fetal genes determined by quantitative real-time PCR indicates upregulation of *nppa* (**G**), *nppb* (**H**), *rcan1.4* (**I**), β-myosin heavy chain (*myh7*) (**J**), and downregulation of α-myosin heavy chain (*myh6*) (**K**) due to AngII treatment; HectD3 overexpression again led to opposite or nullifying effect of AngII. (n=6 (AAV-Luc PBS), 7 (AAV-HectD3 PBS), 11 (AAV-Luc AngII), 11 (AAV-HectD3 AngII)). Statistical calculations were carried out by two-tailed Student’s t-test or by two-way ANOVA. *: p<0.05, **: p<0.01, ***: p<0.001, ns, non-significant.

**
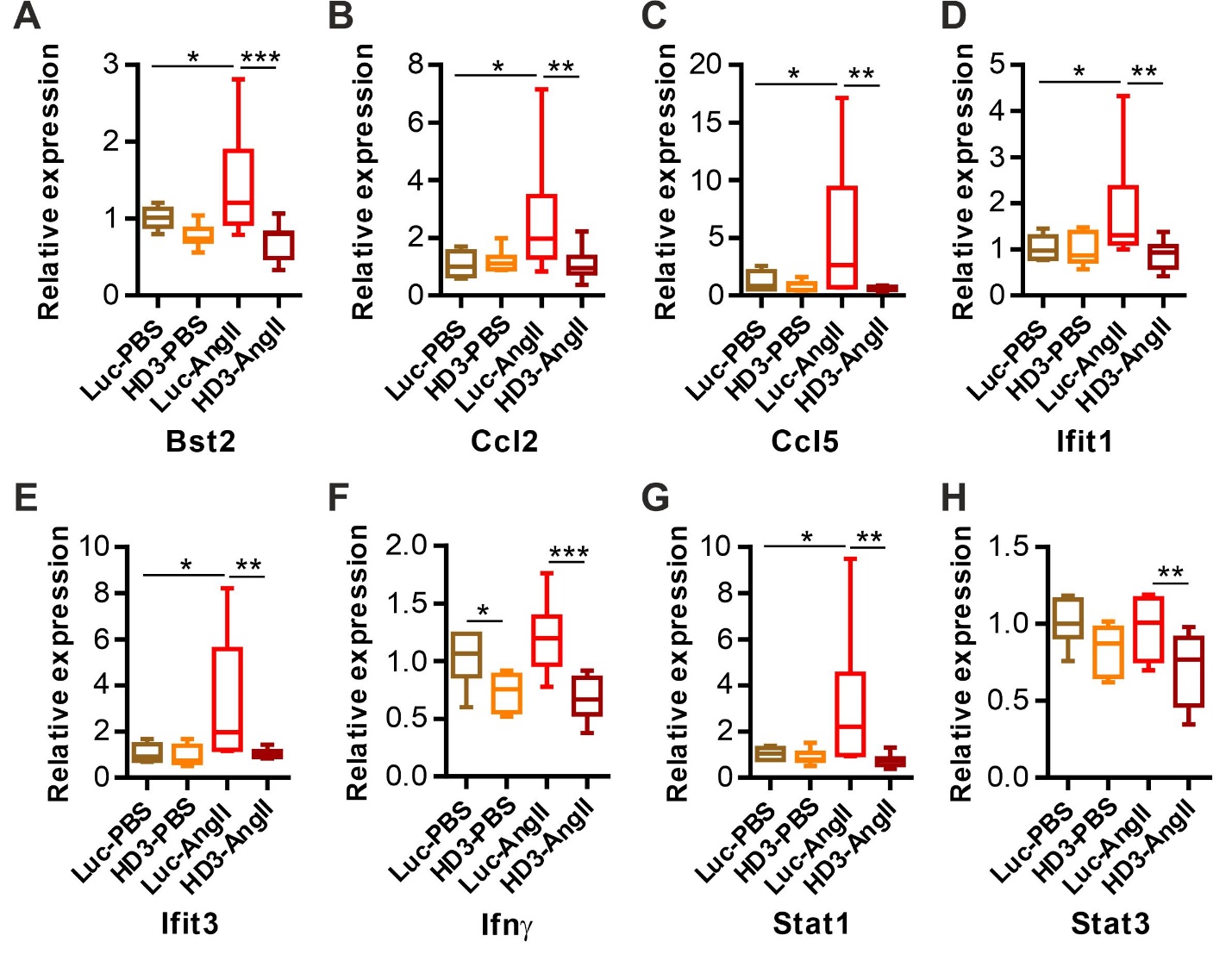
**

**Supplementary Figure 6: HectD3 attenuates Stat1 mediated inflammatory response *in vivo*. A-H.** Transcript levels of some of the inflammatory markers and downstream targets (Bst2, Ccl2, Ccl5, Ifit1, Ifit3, IFNγ, Stat1 and Stat3) of interferon- and Stat1-signaling are detected by quantitative real-time PCR in mouse heart after AngII treatment where HectD3 was overexpressed by AAV-mediated gene transfer. AngII treatment strikingly upregulated expression of these genes. The presence of overexpressed HectD3 again proved to be inhibitory. (n=6 (AAV-Luc PBS), 7 (AAV-HectD3 PBS), 11 (AAV-Luc AngII), 11 (AAV-HectD3 AngII)). Statistical calculations were carried out by two-way ANOVA. *: p<0.05, **: p<0.01, ***: p<0.001.

**
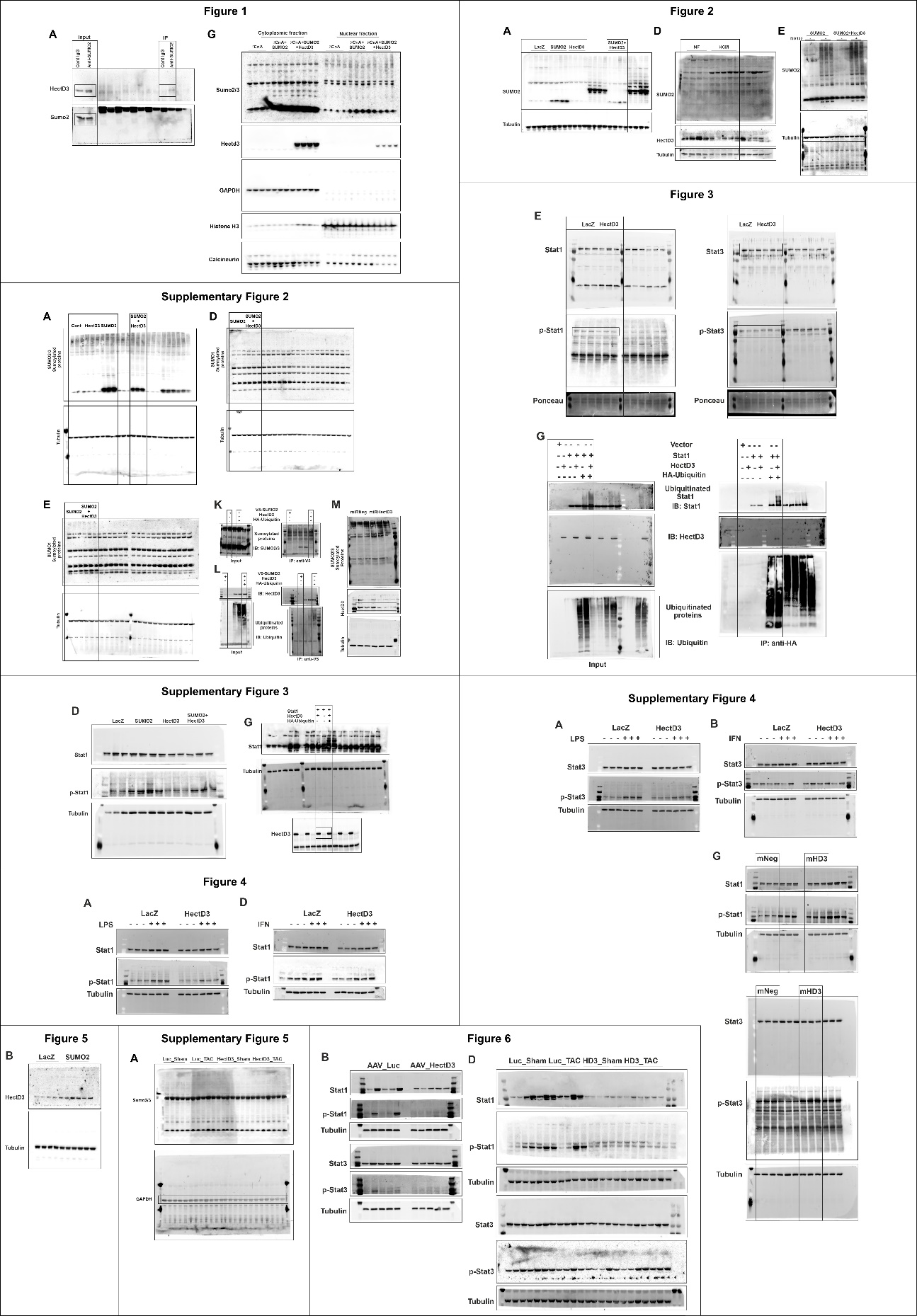
Supplementary Figure 7: Uncropped immunoblot images for the data presented in the main or supplementary figures.**
