# Supplementary material for: The E3 ubiquitin ligase HectD3 attenuates cardiac hypertrophy and inflammation in mice
Source: Commun Biol. 2020 Oct 9;3:562. doi: 10.1038/s42003-020-01289-2 (PMC7547098; doi:10.1038/s42003-020-01289-2)
Supplement: Supplementary file 2 — Description of Additional Supplementary Files [file 42003_2020_1289_MOESM2_ESM.pdf]

Description of Additional Supplementary Files

File Name: Supplementary Data 1
Description: Source data of the graphs presented in main figures

File Name: Supplementary Data 2
Description: Sequence details of the oligonucleotides used in this study
