# Supplementary material for: The E3 ubiquitin ligase HectD3 attenuates cardiac hypertrophy and inflammation in mice
Source: Commun Biol. 2020 Oct 9;3:562. doi: 10.1038/s42003-020-01289-2 (PMC7547098; doi:10.1038/s42003-020-01289-2)
Supplement: Supplementary file 6 — Reporting Summary [file 42003_2020_1289_MOESM6_ESM.pdf]

natureresearch

Corresponding author(s): Ashraf Y Rangrez & Norbert Frey

Last updated by author(s): Aug 31, 2020

Reporting Summary

Nature Research wishes to improve the reproducibility of the work that we publish. This form provides structure for consistency and transparency in reporting. For further information on Nature Research policies, see Authors & Referees and the Editorial Policy Checklist .

Statistics

For all statistical analyses, confirm that the following items are present in the figure legend, table legend, main text, or Methods section.

n/a	Confirmed	
☐	☒	The exact sample size (*n*) for each experimental group/condition, given as a discrete number and unit of measurement
☐	☒	A statement on whether measurements were taken from distinct samples or whether the same sample was measured repeatedly
☐	☒	The statistical test(s) used AND whether they are one- or two-sided<br>*Only common tests should be described solely by name; describe more complex techniques in the Methods section.*
☐	☒	A description of all covariates tested
☐	☒	A description of any assumptions or corrections, such as tests of normality and adjustment for multiple comparisons
☐	☒	A full description of the statistical parameters including central tendency (e.g. means) or other basic estimates (e.g. regression coefficient) AND variation (e.g. standard deviation) or associated estimates of uncertainty (e.g. confidence intervals)
☒	☐	For null hypothesis testing, the test statistic (e.g. *F*, *t*, *r*) with confidence intervals, effect sizes, degrees of freedom and *P* value noted<br>*Give P values as exact values whenever suitable.*
☒	☐	For Bayesian analysis, information on the choice of priors and Markov chain Monte Carlo settings
☒	☐	For hierarchical and complex designs, identification of the appropriate level for tests and full reporting of outcomes
☒	☐	Estimates of effect sizes (e.g. Cohen's *d*, Pearson's *r*), indicating how they were calculated

Our web collection on statistics for biologists contains articles on many of the points above.

Software and code

Policy information about availability of computer code

Data collection	AlphaInnotech Software 3.0.3.0; Keyence BZ-II Viewer; Magellan 7.2 SP1
Data analysis	Bio-Rad CFX Manager 3.1; GraphPad Prism 6; ImageJ 1.4; Keyence BZ-II Analyzer; SigmaPlot 13.0; Vevo LAB 1.7.1

For manuscripts utilizing custom algorithms or software that are central to the research but not yet described in published literature, software must be made available to editors/reviewers. We strongly encourage code deposition in a community repository (e.g. GitHub). See the Nature Research guidelines for submitting code & software for further information.

Data

Policy information about availability of data

All manuscripts must include a data availability statement. This statement should provide the following information, where applicable:

- Accession codes, unique identifiers, or web links for publicly available datasets
- A list of figures that have associated raw data
- A description of any restrictions on data availability

The RNA-sequencing data was deposited to the GEO (Gene Expression Omnibus) database (https://www.ncbi.nlm.nih.gov/geo/) and is available under the accession number GSE155768. The mass spectrometry proteomics data have been deposited to the ProteomeXchange Consortium via the PRIDE partner repository with the dataset identifier PXD020843.

Field-specific reporting

Please select the one below that is the best fit for your research. If you are not sure, read the appropriate sections before making your selection.

Life sciences study design

All studies must disclose on these points even when the disclosure is negative.

Sample size	Sample sizes of animal experiments were chosen based on prior knowledge of statistical power from previously published experiments or publications in the field. For adequate power, we generally chose a sample size of at least n=6 for physiological experiments and at least n=4 or biochemical experiments, and n=4 for RNA-Seq and Proteomics analyses.
Data exclusions	No data was excluded
Replication	For each series of experiments, all attempts at replication were successful.
Randomization	Animals and cells were randomly assigned to the experimental groups.
Blinding	Investigators were blinded to groups allocation during data collection and data analyses.

Reporting for specific materials, systems and methods

We require information from authors about some types of materials, experimental systems and methods used in many studies. Here, indicate whether each material, system or method listed is relevant to your study. If you are not sure if a list item applies to your research, read the appropriate section before selecting a response.

Materials & experimental systems

n/a	Involved in the study	
☐	☒	Antibodies
☐	☒	Eukaryotic cell lines
☒	☐	Palaeontology
☐	☒	Animals and other organisms
☒	☐	Human research participants
☒	☐	Clinical data

Methods

n/a	Involved in the study	
☑	☐	ChIP-seq
☑	☐	Flow cytometry
☑	☐	MRI-based neuroimaging

Antibodies

Antibodies used

α-actinin (EA-53, lot #:127M4807V), mouse monoclonal, Sigma-Aldrich (1:400); Catalog #: A7811
SUMO1 (lot #:1), rabbit polyclonal, Cell-signaling (1:1000); Catalog #: 4930
SUMO2/3 (18H8, lot #:6), rabbit monoclonal, Cell signaling (1:1000); Catalog #: 4971
SUMO2+3 (8A2, lot #:GR3192502-1), mouse monoclonal, Abcam, (1:1000); Catalog #: ab81371
Calcineurin A (29/Calcineurin, lot #:4171969), mouse monoclonal, BD Bioscience (1:250); Catalog #: 610260
GAPDH (GAPDH-71.1, lot #:039M4772V), mouse monoclonal, Sigma-Aldrich (1: 20000); Catalog #: G8795
Histone H3 (D1H2, lot #:9), rabbit polyclonal, Cell-signaling (1:1000); Catalog #: 4499
α-Tubulin (B-5-1-2, lot #:039M4769V), mouse monoclonal, Sigma-Aldrich (1:8000); Catalog #: T5168
Ubiquitin (FK2, lot #:2819680), mouse monoclonal, Millipore (1:1000); Catalog #: 04-263
STAT1 (D1K9Y, lot #:4), rabbit monoclonal, Cell signaling (1:1000); Catalog #: 14994
p-STAT1 (Tyr701, lot #:23), rabbit monoclonal, Cell signaling (1:1000); Catalog #: 9167
STAT3 (lot #:7), rabbit monoclonal, Cell signaling (1:1000); Catalog #: 9132
p-STAT3 (Tyr705, lot #:8), rabbit monoclonal, Cell signaling (1:1000); Catalog #: 9145
F4/80 (BM8, lot #:016689), rat monoclonal, Dianova (1:500);
HA (HA-7, lot #:112B4841), mouse monoclonal, Sigma-Aldrich (1:20000); Catalog #: H9658
HectD3 (lot #:AK0017MAY19093), rabbit polyclonal, Mybiosource (1:1000); Catalog #: MBS2519007
V5 (SV5-P-K, lot #:T1521A06), mouse monoclonal, Biozol (1:000); Catalog #: Ab00136-3.0

Validation

Validated by manufacturer or by using overexpression constructs.

Eukaryotic cell lines

Policy information about cell lines

Cell line source(s)	HEK293 (ATCC, CRL-1573)
Authentication	None of the cell lines used were authenticated

Mycoplasma contamination	None
Commonly misidentified lines (See ICLAC register)	No cell line is listed by ICLAC

Animals and other organisms

Policy information about studies involving animals; ARRIVE guidelines recommended for reporting animal research

Laboratory animals	Animals in this study had the genetic backgounds C57BL/6J as indicated. For neonatal rat cardiomyocytes, pups of Sprague Dawley rats were used
Wild animals	None
Field-collected samples	None
Ethics oversight	All animal experiments were approved by the Ministry of Energy Transition, Agriculture, Environment, Nature and Digitalization (MELUND) of the state of Schleswig-Holstein and were carried out stringently following international and institutional ethical guidelines.

Note that full information on the approval of the study protocol must also be provided in the manuscript.

ChIP-seq

Data deposition

☐ Confirm that both raw and final processed data have been deposited in a public database such as GEO.

☐ Confirm that you have deposited or provided access to graph files (e.g. BED files) for the called peaks.

Data access links *May remain private before publication.*	*For "Initial submission" or "Revised version" documents, provide reviewer access links. For your "Final submission" document, provide a link to the deposited data.*
Files in database submission	*Provide a list of all files available in the database submission.*
Genome browser session (e.g. UCSC)	*Provide a link to an anonymized genome browser session for "Initial submission" and "Revised version" documents only, to enable peer review. Write "no longer applicable" for "Final submission" documents.*

Methodology

Replicates	*Describe the experimental replicates, specifying number, type and replicate agreement.*
Sequencing depth	*Describe the sequencing depth for each experiment, providing the total number of reads, uniquely mapped reads, length of reads and whether they were paired- or single-end.*
Antibodies	*Describe the antibodies used for the ChIP-seq experiments; as applicable, provide supplier name, catalog number, clone name, and lot number.*
Peak calling parameters	*Specify the command line program and parameters used for read mapping and peak calling, including the ChIP, control and index files used.*
Data quality	*Describe the methods used to ensure data quality in full detail, including how many peaks are at FDR 5% and above 5-fold enrichment.*
Software	*Describe the software used to collect and analyze the ChIP-seq data. For custom code that has been deposited into a community repository, provide accession details.*

Flow Cytometry

Plots

Confirm that:

☐ The axis labels state the marker and fluorochrome used (e.g. CD4-FITC).

☐ The axis scales are clearly visible. Include numbers along axes only for bottom left plot of group (a 'group' is an analysis of identical markers).

☐ All plots are contour plots with outliers or pseudocolor plots.

☐ A numerical value for number of cells or percentage (with statistics) is provided.
